# Animal- and Plant-Derived Protein Nanocarriers for the Delivery of Natural Compounds in Breast Cancer Chemoprevention

**DOI:** 10.3390/molecules31132391

**Published:** 2026-07-07

**Authors:** Zuzanna Senkowska, Julia Wojtkowicz, Dominik Zakrzewski, Katarzyna Owczarek, Karolina Niewinna, Urszula Lewandowska

**Affiliations:** 1 Department of Biochemistry, Faculty of Medicine, Medical University of Lodz, 92-215 Lodz, Poland; zuzanna.senkowska@umed.lodz.pl (Z.S.); dominik.zakrzewski2@student.umed.lodz.pl (D.Z.); katarzyna.owczarek@umed.lodz.pl (K.O.); karolina.niewinna@umed.lodz.pl (K.N.); 2 Department of Medical Biology, Faculty of Pharmacy, Medical University of Lodz, 90-151 Lodz, Poland; julia.wojtkowicz@umed.lodz.pl

**Keywords:** bioavailability, breast cancer, chemoprevention, encapsulation, nanocarriers, polyphenols, protein-based nanocarriers

## Abstract

Breast cancer remains one of the leading causes of cancer-related mortality among women worldwide, highlighting the need for safer and more effective chemopreventive strategies. Although many phytochemicals can modulate key molecular processes involved in breast carcinogenesis, their chemopreventive potential largely depends on delivery strategies that preserve their biological activity and enable efficient accumulation at the target site. Protein-based nanocarriers have emerged as promising delivery systems capable of improving the protection, solubility, cellular uptake, targeted delivery, and controlled release of bioactive compounds in tumor tissues. This review summarizes recent advances in selected animal- and plant-derived protein nanocarriers used for the encapsulation and delivery of natural compounds in breast cancer chemoprevention. Particular attention is given to their physicochemical properties, encapsulation performance, release behavior, biological activity, targeting potential, and translational limitations. Furthermore, the mechanisms underlying the enhanced anticancer activity of encapsulated phytochemicals, including improved stability, receptor-mediated uptake, pH-responsive release, apoptosis induction, oxidative stress modulation, and inhibition of tumor growth and metastasis, are highlighted. Current challenges, including enzymatic degradation, formulation instability, immunogenicity concerns, manufacturing scalability, and limited clinical evidence, remain important barriers to translation. Overall, selected protein-based nanocarriers represent promising multifunctional platforms for improving the chemopreventive potential of natural compounds in breast cancer.

## 1. Introduction

Malignant neoplasms represent a significant barrier to increasing the average life expectancy and remain the leading cause of premature mortality globally, demonstrating a consistent rising trend [1]. Among all types of cancer, breast cancer (BC) accounts for a substantial proportion of cases and remains one of the major challenges for healthcare systems around the world. It is estimated that 2.3 million new cases were diagnosed in 2022, representing 25% of all newly diagnosed cancers. Additionally, 670,000 deaths from this disease were recorded, representing 15.5% of cancer-related deaths among females. Furthermore, even when both females and males are considered, BC remains the second most frequently diagnosed cancer type and the fourth most common cause of cancer-related mortality [2].

Despite the considerable advances that have already been achieved in treating this disease, the above-mentioned statistics indicate that further improvements in therapy are still needed. BC is commonly managed using multimodal treatment, including surgical procedures, radiotherapy, chemotherapy, hormone treatment, and molecularly targeted therapy [3]. However, the increasing prevalence of multidrug resistance (MDR) and systemic toxicity associated with standard oncological therapies is becoming increasingly evident. Therefore, modern oncology is also moving toward the use of naturally derived compounds to support standard treatment. Notably, nearly 50% of drugs introduced in recent decades are derived from or inspired by natural substances [4,5].

Chemoprevention, defined as the use of natural agents to inhibit, reverse, or delay carcinogenesis at various stages, is a critical strategy for reducing cancer incidence [6]. This approach involves a wide range of biologically active phytochemicals found in plants [6,7]. For example, curcumin (CUR) and resveratrol, which are known for their ability to modulate signaling pathways, inhibit cell proliferation, induce apoptosis, and increase the generation of reactive oxygen species (ROS), have been widely investigated in this context [8,9]. Flavonoids, such as quercetin, kaempferol, and epigallocatechin gallate (EGCG), exhibit anti-inflammatory and antioxidant properties that inhibit BC cell proliferation [9]. Among the alkaloids, berberine, in addition to the aforementioned effects, can also arrest the cell cycle and inhibit angiogenesis and metastasis [10]. Terpenoids, such as artemisinin, may induce ferroptosis through ROS generation. Natural products also play an important role in combination therapies by enhancing tumor sensitivity to Food and Drug Administration (FDA)-approved agents, inhibiting immune checkpoints, and protecting healthy tissues against chemotherapy-induced toxicity [11]. Studies have confirmed that CUR may act as a chemosensitizer in BC treatment when combined with doxorubicin (DOX), enhancing its cytotoxic and tumor growth inhibitory effects while simultaneously protecting normal cells [12].

Despite their therapeutic potential, the clinical application of phytochemicals remains limited due to poor water solubility, low bioavailability, rapid metabolism, insufficient gastrointestinal absorption, and instability under physiological conditions [13,14,15]. Moreover, many natural compounds are highly sensitive to environmental factors such as pH, light, oxygen, enzymes, and temperature [16]. Anthocyanins represent a notable example, as their stability is strongly dependent on pH conditions and decreases significantly at neutral and alkaline pH levels [17]. These limitations significantly reduce the therapeutic effectiveness of natural compounds and highlight the need for advanced delivery systems capable of improving their stability and biological activity.

To overcome these obstacles, encapsulation-based delivery systems have been proposed as an effective strategy to enclose active substances within protective carriers, thereby shielding them from degradation and enabling their controlled release at the target site. Among the various biomaterials used for encapsulation, proteins have attracted particular attention owing to their biocompatibility, biodegradability, structural versatility, and ability to interact with bioactive molecules through hydrophobic, electrostatic, and hydrogen-bonding interactions. These features make protein-based nanocarriers especially promising platforms for improving the delivery and chemopreventive potential of natural compounds in BC.

## 2. Protein-Based Nanocarriers

The use of proteins as building materials for encapsulation enables the targeted delivery of anticancer agents to tumors, which is essential for improving therapeutic efficacy while reducing systemic toxicity. Nano-based drug delivery systems achieve this through both passive and active targeting mechanisms [18]. Passive targeting relies mainly on the enhanced permeability and retention effect, which enables nanoparticle (NP) accumulation within tumor tissues due to abnormal tumor vasculature and impaired lymphatic drainage [19,20]. Tumor microvessels possess high permeability, with pore diameters ranging from 100 nm to 2 µm, facilitating nanocarrier extravasation and prolonged retention in the tumor microenvironment [18]. In contrast, active targeting is based on specific ligand–receptor interactions between functionalized nanocarriers and receptors overexpressed on cancer cells [21].

A wide range of delivery systems have been developed, including organic and inorganic nanocarriers [22]. Polysaccharide-based systems, such as chitosan and pectins, exhibit high biocompatibility and protect cargo against digestive enzymes [23]. Lipid-based carriers, including liposomes, improve compound permeability across biological barriers and reduce oxidative degradation [24]. Inorganic nanocarriers, such as silica- and metal-based NPs, provide high mechanical strength and stability, minimizing premature release and improving resistance to environmental stressors [25].

Among the available delivery platforms, protein-based nanocarriers have gained particular attention due to their biodegradability, biocompatibility, edibility, digestibility, and structural versatility [26]. Proteins can be fabricated into diverse nanostructures, including spheres, fibers, tubes, hydrogels, and micelles, depending on the intended biomedical application. Furthermore, protein carriers may protect encapsulated phytochemicals against oxidative degradation and improve their stability and bioavailability. Their abundant functional groups also facilitate interactions with other biopolymers and enable surface functionalization for active targeting [26].

Protein nanocarriers have been extensively investigated in oncology, including BC therapy and chemoprevention. Among them, animal-derived proteins, such as albumin, gelatin, casein, and silk fibroin, and plant-derived proteins, such as zein, glutenin, and gliadin, and ferritin—which can be obtained from both animal and plant sources—represent the most commonly explored systems for the encapsulation and targeted delivery of natural compounds [18,27,28]. These nanocarriers exhibit distinct physicochemical properties, encapsulation efficiencies, release profiles, and biological activities that may significantly improve the therapeutic potential of phytochemicals in BC models. When compared with conventional synthetic polymers and lipid-based carriers, protein-based systems may offer advantages in terms of cellular absorption capabilities, low antigenicity, and non-toxic metabolic end products. Furthermore, they offer enhanced long-term storage stability compared with alternative drug delivery systems. However, each system also presents specific limitations that should be considered during formulation design and future clinical translation. It is worth mentioning that natural proteins are associated with relatively high manufacturing costs, since the process involves several complicated stages of isolation and purification. Additionally, biopolymers may exhibit poor colloidal stability because of the sensitivity of their highly dynamic secondary structures to changes in pH and temperature [29,30].

This review focuses on the molecular characteristics, physicochemical properties, and anticancer activities of natural compounds encapsulated within protein-based NPs, particularly in the context of BC chemoprevention. The advantages and current limitations associated with each protein-based delivery system are also discussed.

## 3. Animal-Derived Protein Nanocarriers

Given their availability, promising biological properties, biocompatibility, and nutritional origin [31,32], animal-derived protein-based NPs have proven to be promising carriers for natural compounds in BC. Specifically, albumin, gelatin, casein, and silk fibroin possess unique characteristics that make them potential platforms for enhancing the bioavailability and efficacy of phytochemicals in BC models. Indeed, all of these biomolecules have distinctive properties related to their molecular weight, isoelectric point, solubility, and structure, which together determine the physicochemical and functional properties of the resulting carriers. The main properties of these systems are presented in Table 1.

### 3.1. Albumin-Based Nanocarriers

Albumin is an animal-derived protein that has attracted considerable scientific interest as a biomolecule widely used in targeted drug delivery. There are many types of albumins that have been extracted and utilized for biomedical studies including: human serum albumin (HSA), bovine serum albumin (BSA), ovalbumin, which is derived from egg white, and rat serum albumin. Mammalian albumins constitute a family of globular, water-soluble proteins synthesized in the liver and represent the most abundant class of proteins in human blood plasma, with concentrations of approximately 35–50 g/L. Structurally, albumin is a heart-shaped molecule with a molecular weight of 65–70 kDa and a half-life of 19 days. Albumin is stable at pH 4–9 and shows thermal resistance after exposure to 60 °C for 10 h. Among the different albumin types, two are used most frequently in biomedical applications: BSA and HSA. HSA comprises a single polypeptide chain of 585 amino acid residues and has a molecular weight of 66.5 kDa. It shows high cysteine and low tryptophan content [33,34]. BSA, on the other hand, weighs approximately 69 kDa and consists of 583 amino acid residues [35]. Native BSA contains multiple disulfide bonds that function as intrinsic cross-linking elements. These bonds are cleaved under reducing conditions and can be re-established when exposed to an oxidizing environment [37]. Both proteins have similar isoelectric points of approximately 4.7 [36]. Several studies have shown that albumin can act as a suitable carrier for drug attachment and delivery due to its nontoxic, fully biodegradable, and biocompatible nature [34,50,51]. Furthermore, there are no sugar residues attached to albumins, which makes them less immunogenic. Albumin is readily available in vertebrate blood plasma, where it represents about 60% of serum proteins, which supports its use as a carrier that can be administered in relatively large amounts with a favorable safety profile. From an economic point of view, the high abundance of albumins and their relatively simple and low-cost production make them highly advantageous for pharmaceutical applications, as lower manufacturing expenses contribute to making medications more affordable for both healthcare systems and patients [52].

All of these features have been utilized to develop albumin-based NPs that function as delivery platforms for anticancer drugs. A notable early example is HSA nanoparticles loaded with paclitaxel, marketed as Abraxane, which received approval from the FDA for the treatment of metastatic BC [53]. This represents a clinically approved example of the albumin-based delivery of a synthetic anticancer compound in BC therapy. Nevertheless, the lack of selectivity between healthy and cancerous cells, along with the development of MDR, remains a major limitation of conventional chemotherapeutics. Therefore, there is a continuing need for new strategies and innovative chemopreventive agents. Phytochemicals obtained from medicinal plants have emerged as promising candidates for BC therapy, and the use of advanced delivery systems to improve their bioavailability and enable targeted drug delivery is increasingly important [54]. In vitro studies using BC cell lines including MDA-MB-231 and MCF-7 demonstrate the effective anticancer properties of naturally derived compounds [55,56,57]. However, encapsulation of these compounds in albumin-based carriers may further enhance their biological activity. Evodiamine, a natural indolequinone alkaloid isolated from *Evodia rutaecarpa*, shows markedly improved cellular uptake when encapsulated in BSA to form evodiamine-loaded nanoparticles (ENPs) compared with the free compound. Enhanced uptake leads to a significantly greater reduction in cell viability than that observed with the unencapsulated compound. This suggests that encapsulation improves the bioavailability of natural compounds. In addition, ENPs more effectively suppress colony formation and display stronger pro-apoptotic activity. Both free evodiamine and ENPs upregulate the expression of p53, caspase-7, caspase-9, and Bax, while downregulating Bcl-2. However, ENPs result in a significantly greater upregulation of cell death-promoting markers and a more pronounced downregulation of mRNA expression associated with anti-apoptotic activity compared with the free compound. While evodiamine induces cell cycle arrest at the G2/M phase, encapsulation in BSA NPs further increases intracellular delivery, resulting in a higher proportion of cells arrested in this phase (in MDA-MB-231 cells, 15.5% of evodiamine-treated cells and 19.7% of ENP-treated cells were arrested in G2/M, while in MCF-7 cells, the values were 23.6% and 62.0%, respectively). The enhanced anticancer activity of ENPs is also reflected in their stronger induction of apoptosis [54]. There are also other examples of using a protein coating to enhance the anticancer effect of a natural extract. Plumbagin, a naturally occurring phytochemical, mainly found in the roots of the *Plumbago zeylanica* L., also exhibits significantly better cytotoxic activity against BC cells, reduces colony formation and increases cellular uptake compared with the free extract when encapsulated in a BSA carrier [58]. The use of a protein carrier also increases the physical stability of the natural extract, making its action more resistant to external factors, such as temperature or pH [59].

The effectiveness of the encapsulated extract depends on releasing it at the appropriate site, for a sufficient duration, and in an efficient manner [59]. The extracellular pH, which is maintained at 7.4 in normal tissues, decreases to approximately 6.8 in tumors. This phenomenon has also been observed in cases of BC [60]. Thus, for the successful release of the natural compound, the delivery vehicle should enable pH-dependent release of the compound. This requirement aligns with another property of BSA, which has been shown to release the extract significantly more efficiently in an acidic environment than under neutral conditions [54,61].

Moreover, BSA demonstrates greater release efficiency for the natural compound than polysaccharide carriers such as cellulose or chitosan. In research on the anticancer effect of proteins loaded with carnosic acid against BC, the protein shell showed better release performance within ten hours. Approximately 75% of the encapsulated compound was released from the albumin shell, compared with 61% from chitosan NPs and 15.6% from cellulose [59]. Modifications of NPs, especially through proper NP selection, have proven to be useful for improving drug entrapment and minimizing drug toxicity by facilitating drug accumulation in target tissues or organs. Modification also allows for better dosage adjustment and minimization of adverse reactions [62]. Albumin NPs offer excellent potential for surface modification due to the presence of many reactive functional groups, such as amino, carboxyl, and thiol groups, enabling easy covalent modification and active targeting [52]. The most popular surface modification technique is PEGylation, which involves the covalent or non-covalent attachment of polyethylene glycol (PEG) to a protein molecule. By adding PEG, the hydrodynamic volume of the biomolecule increases, thereby creating a protective steric barrier. This leads to lower levels of renal clearance, extending the blood circulation time of the biomolecule in direct proportion to the molecular weight of PEG [63]. Encapsulation of CUR into PEG-albumin NPs has been reported to improve antiproliferative activity against the MCF-7 cell line as well as decrease the uptake by liver and Kupffer cells when compared with CUR-albumin NPs. This demonstrates the stealth characteristics of PEGylated encapsulation systems [64]. Albumin-based encapsulation may enhance cytotoxic selectivity toward tumor cells while sparing normal cells, owing to its innate in vivo tumor tropism, making it an ideal cancer drug carrier [53,65]. In addition to PEGylation, folic acid (FA) conjugation is an extensively used surface modification technique. The FA has several advantages, including high stability, low immunogenicity, and high affinity for folate receptor alpha (FRα), which is overexpressed in many cancer cells compared with normal cells. FA-modified BSA NPs have the capacity to interact with the FRα and enter the cells through receptor-mediated endocytosis. Thus, the NPs accumulate more in tumors, target cancerous cells, and minimize adverse effects on healthy cells [61]. This strategy has also been applied in BC chemoprevention. FA conjugated to BSA-PEG NPs loaded with naringenin showed no toxicity toward normal fibroblasts, while exhibiting concentration-dependent cytotoxicity toward MCF-7 cells [62].

Research on the effectiveness of albumin-based NPs extends beyond in vitro studies to animal models, including mice [66] and rats [67]. In vivo experiments provide additional insight into drug release and tissue uptake profiles. For instance, CUR enclosed in albumin NPs, administered intravenously to Wistar rats, has been shown to protect the compound in systemic circulation, thereby improving its dissolution rate and enhancing bioavailability. As a result, higher drug concentration can be achieved at the target site, which is reflected in increased systemic drug levels observed for NP formulations [67]. In vivo studies enable the direct evaluation of tumor size following treatment. Experiments conducted on female BALB/c mice demonstrated a reduction in tumor size in treated groups compared with untreated animals. Furthermore, when the active compound, anethole, was encapsulated in albumin NPs modified with FA-conjugated chitosan and administered via intraperitoneal injection, it demonstrated significantly stronger anticancer properties compared with the free extract alone. Tumor growth inhibition after 15 days of treatment was 36.9% for the free compound and 56.6% and 64.9% for the NPs at 25 and 50 mg/kg/tumor volume, respectively. These findings suggest that the encapsulated formulation is more effective at inhibiting tumor growth during therapy [66].

Potential limitations of albumin-based NPs include their susceptibility to degradation in the gastrointestinal tract following oral administration. Proteolytic enzymes such as pepsin and trypsin may alter BSA, thereby limiting its effectiveness as a carrier for bioactive compounds. To address this issue, antitumor formulations incorporating carboxymethyl pachymaran have been proposed; for example, CUR-loaded BSA/carboxymethyl pachymaran particles have been developed to improve resistance to gastrointestinal degradation [68]. Other challenges in translating this technology into clinical applications include scaling up, batch-to-batch differences, and the possible toxicity caused by using cross-linkers in the synthesis of BSA NPs [69]. Although many studies found no acute toxicological effects, the possible consequences of chronic exposure to the particles remain insufficiently investigated [70]. Therefore, despite highly promising results in cellular and animal models, several aspects still require further investigation before widespread clinical application can be achieved.

### 3.2. Gelatin-Based Nanocarriers

Another promising material in bioactive compound delivery systems is gelatin. It is becoming increasingly popular because of its biocompatibility, biodegradability, and low immunogenicity [71]. As a product of partial collagen hydrolysis, it can be formulated into gels, hydrogels, microcapsules, and nanocarriers, making it a versatile platform for the encapsulation of active substances [72,73].

Gelatin is a natural protein-based biopolymer obtained through the partial hydrolysis of collagen derived from animal tissues such as the skin, bones, tendons, and cartilage of bovine, porcine, fish, and poultry origin [72]. The properties of the final product depend on the source material, type of tissue, age of the animal, and extraction conditions, which may contribute to considerable batch-to-batch variability [74]. Gelatin is most commonly produced via the acid, alkaline, or thermal hydrolysis of collagen [38]. Therefore, it is also referred to as hydrolyzed collagen or collagen peptides [75]. Structurally, gelatin retains the characteristics of a fibrous protein closely related to collagen [39]. Its polypeptide chains are based on a repeating sequence motif of the type (Gly-X-Pro)_n_, where glycine is the predominant amino acid, while the other position is frequently occupied by proline and hydroxyproline [40]. Variations in polypeptide chain length and degree of hydrolysis significantly influence the mechanical and functional properties of the material [38]. Although this heterogeneity may represent a limitation, it simultaneously allows the gelatin properties to be tuned, so that the gelatin properties meet specific biomedical application requirements.

In technological applications, two primary types of gelatin are of particular importance. Type A gelatin, predominantly obtained via acid treatment, is characterized by a higher isoelectric point (approximately pH 9) and a more cationic nature. In contrast, Type B gelatin is typically produced through alkaline hydrolysis and exhibits a lower isoelectric point (approximately pH 5), resulting in a more anionic character [41]. Type B gelatin usually exhibits a molecular weight in the range of 40–90 kDa, whereas type A gelatin shows a molecular weight of approximately 90–100 kDa [38]. These differences significantly influence electrostatic interactions with encapsulated molecules, the colloidal stability of the systems, and the release profile of active substances [74,76].

Gelatin dissolves in water upon heating and undergoes reversible gelation during cooling. Its viscosity and ability to form a three-dimensional network depend on temperature, pH, concentration, and the ionic strength of the environment [76]. These thermoresponsive properties facilitate material processing, particle formation, and the encapsulation of bioactive compounds within the polymeric matrix [77,78]. Additionally, gelatin can be used to fabricate scaffolds with an open, porous structure, whose architecture can be tailored according to the intended biomedical application [77].

A significant advantage of gelatin is its ability to be combined with other polymers or ligands, enabling the design of advanced multifunctional systems [79]. This feature enables the development of stimuli-responsive delivery platforms that react to changes in pH, temperature, or the presence of enzymes, particularly matrix metalloproteinases (MMPs), whose expression is elevated in the tumor microenvironment [76].

In the context of BC chemoprevention, particular attention is given to its ability to enhance the stability, solubility, and controlled release of natural compounds, which often exhibit limited bioavailability in their free form [80]. Encapsulation within a gelatin matrix can reduce their degradation, improve stability, and enable controlled release, thereby enhancing the biological efficacy of the formulation [25].

The effectiveness of gelatin as an encapsulation material has been demonstrated in numerous studies related to BC therapy. Gelatin-based lipid nanocarriers encapsulating *Berberis aristata* extract (GLN-BA) show promising anticancer potential, as evidenced by clear quantitative data. Under in vitro conditions, GLN-BA displayed a 2-fold lower IC50 against MCF-7 cells (4.73 ± 2.95 μg/mL) than the free extract (10.18 ± 3.04 μg/mL), reflecting a substantial increase in cytotoxicity. Flow cytometry revealed 18.54% apoptotic cells and 27.31% in sub-G1 after GLN-BA treatment, versus 8.67% apoptotic cells for the free extract, indicating intensified apoptosis-driven tumor cell death [81]. Similarly, a gelatin-based formulation in which the ethyl acetate extract from Cantigi (*Vaccinium varingiaefolium*) leaves was loaded into gelatin nanoparticles (GNPs) also improved the anticancer activity against T47D breast cancer cells. The crude extract showed an IC_50_ of 75.16 ± 3.05 μg/mL, whereas the optimized GNP-EA formulation reduced this value to 16.88 ± 1.42 μg/mL, indicating about a 4.5-fold increase in cytotoxic potency [82]. Gelatin has also been used as a component of injectable oxidized alginate-gelatin hydrogels for the combined delivery of DOX and quercetin. In MCF-7 cells, the hydrogel containing DOX alone increased the cytotoxic activity 4.66-fold compared with free DOX, whereas the dual-delivery formulation containing both DOX and quercetin-loaded nanoparticles enhanced the cytotoxicity 20.7-fold, highlighting the potential of combination therapy and gelatin-based injectable systems for BC treatment [83].

Additionally, PEGylated gelatin nanoparticles co-loading DOX and betanin (BET) demonstrate strong potential for anticancer therapy, enabling the efficient delivery of both agents to tumor cells, pH-triggered controlled release, and enhanced cytotoxicity against MCF-7 cells. The system achieved high encapsulation efficiencies (82% for DOX and 65% for BET) and drug loading of 20.5% (DOX) and 16.25% (BET), and showed markedly greater release under acidic, tumor-like conditions (approximately 81% DOX and 50% BET released after 48 h at pH 5.3) compared with physiological pH (about 24% DOX and 18% BET released after 48 h at pH 7.4) [84].

Gelatin also plays a significant role in encapsulating natural compounds. CUR, which is characterized by poor solubility and low bioavailability, has demonstrated enhanced stability, improved cellular uptake, increased antiproliferative and cytotoxic effects, including induction of cancer cells death, against BC cells following encapsulation in gelatin-based nanogels or nanoemulsions, as observed in the MCF-7 [85] and MDA-MB-231 cell lines [86]. Oleic acid-conjugated gelatin NPs (GONs) with sesamol are another example of using gelatin as a carrier for a natural compound. Sesamol is a phenolic compound that is typically found in the seeds of *Sesamum indicum*. It is currently regarded as a dietary antioxidant. Oleic acid had minimal influence on the in vitro release of sesamol from NPs. GONs showed a biphasic release pattern, characterized by an initial burst within the first 2 h due to surface-adsorbed sesamol, followed by sustained release from the entrapped compound. Sesamol-loaded GONs significantly reduced the MCF-7 cell viability, with an IC_50_ value significantly lower than that of free sesamol. In contrast, plain GONs showed no detectable cytotoxicity, confirming their biocompatibility and safety [87]. Gelatin can also be used to modify existing carriers to enhance their performance. For example, betulinic acid encapsulated in gelatin–γ-cyclodextrin nanoparticles (BA/GGNPs) demonstrated stronger antitumor activity in BC cells than betulinic acid/γ-cyclodextrin (BA/GCD), likely due to dual-targeting functional groups. These NPs prolonged drug release, enabling sustained exposure and continuous action against tumor cells. As a result, BA/GGNPs showed superior efficacy compared with free BA and BA/GCD, while maintaining selectivity toward cancer cells without inducing toxicity in normal cells [88]. A further example of the use of a natural compound in combination with a gelatin carrier is EGCG incorporated into gelatin-based NPs that contain a soft gel core along with an attached polyelectrolyte shell. The encapsulation process resulted in significantly decreased release kinetics compared with gelatin NPs without the polyelectrolyte shell, in which EGCG was completely released in less than 15 min, while EGCG reached maximal concentrations after 8 h in the shell-containing formulation. This property allows the active compound to act longer on BC cells, increasing its effectiveness and anticancer activity. EGCG is also known to inhibit numerous signaling pathways linked to BC, such as the c-Met/ Hepatocyte Growth Factor (HGF) pathway. HGF binds the c-Met receptor, resulting in intracellular signaling events responsible for triggering cell motility and invasion processes. EGCG enclosed in gelatin NPs revealed the complete inhibition of this signaling pathway, suggesting its anticancer activity [89].

These findings suggest that gelatin may serve as a valuable platform for the delivery of both conventional chemotherapeutic agents and natural chemopreventive compounds.

Furthermore, in vivo testing confirmed the feasibility of technology for the entrapment of bioactive compounds. In mice bearing HER-2-overexpressing breast tumors, gelatin-based nanocarriers enhanced the anticancer potential of the natural compound CUR by improving its delivery to the tumor site and combining it with DOX. The best formulation, Herceptin-conjugated gelatin–calcium phosphate nanocarrier co-loaded with CUR and DOX (HER-AGIO@CaP-CD) contained 2.6 mg/kg CUR and 1.375 mg/kg DOX, together with approximately 2.2 mg/kg Herceptin, and was administered via the tail vein as 100 μL of a 5 wt.% saline suspension after the tumors had reached approximately 100 mm^3^. Its tumor accumulation was further increased by magnetic targeting applied for 8 h. Thus, the anticancer effect resulted not only from the presence of curcumin itself, but also from its improved release in the tumor environment, higher local concentration, and synergy with DOX, leading to stronger tumor growth inhibition than formulations without magnetic guidance [90].

Promising in vivo results were also obtained using natural compounds alone. For instance, a *Berberis aristata* aqueous extract loaded into gelatin lipid nanocarriers demonstrated notable antitumor activity. In a study conducted on female mice, the formulation showed anticancer effects similar to those produced by cisplatin. By day 10, the extract encapsulated in NPs (10 mg/kg) reduced the tumor volume to 35 ± 11%, while cisplatin (3 mg/kg) achieved a 41 ± 5% reduction. Moreover, molecular modeling confirmed the stability of the obtained NPs containing active agents [81].

The effectiveness of gelatin carriers was also tested for transporting a natural compound and a standard drug used in BC therapy. Gelatin-modified lipid NPs co-loaded with quercetin, docetaxel, and imatinib were tested in 4T1 tumor-bearing BALB/c mice. Tumors were allowed to grow to ~200 mm^3^, after which the NPs were injected via the tail vein (10 mg/kg docetaxel and imatinib; 5 mg quercetin). This reduced the tumor volume to ~35% of the saline control (vs. ~75% for free drugs) and decreased the number of pulmonary metastases [91]. Similarly, gelatin-based NPs incorporating DOX and indocyanine green have also been developed, showing effective inhibition of tumor growth. The nanoparticles were intravenously injected into BALB/c mice once the tumor volumes reached 100–200 mm^3^. This system responded to laser irradiation as well as MMP-2 enzymes present in the tumor microenvironment, enhancing localized drug release and improving therapeutic efficacy in mouse models [92].

Despite promising results obtained in preclinical studies using animal models, the application of gelatin-based delivery systems in clinical oncology remains limited. To date, human studies have primarily focused on the use of gelatin as a component of capsules for natural compounds rather than as an advanced nanosystem for targeted BC therapy. One example is a completed phase II clinical trial involving BC patients, which evaluated the effects of a gelatin-encapsulated extract of American ginseng root on tumor biomarkers and treatment safety when administered for 5–14 days prior to surgery. These findings indicate that the clinical application of gelatin in oncology is still at an early stage of development [93].

Although gelatin has numerous advantages, gelatin also has important limitations. As a material of animal origin, its use may be restricted in cultural or religious contexts in which animal-derived products are prohibited [94]. Additionally, gelatin exhibits limited stability under conditions of elevated temperature and humidity and may undergo liquefaction at temperatures close to physiological levels, which complicates its in vivo application [95]. It is also susceptible to degradation by proteolytic enzymes, including proteases, which can result in the premature release of encapsulated active substances [96]. To improve its stability, crosslinking strategies are commonly employed; however, these modifications may reduce biodegradability, affect biocompatibility, and alter the release profile of the encapsulated compounds [97].

### 3.3. Casein-Based Nanocarriers

Milk proteins, especially casein, possess multifunctional characteristics, such as biodegradability and biocompatibility, which make them suitable for developing NPs designed to protect and deliver bioactive compounds [98]. In the mammary gland, their primary biological role is to provide calcium, phosphate, and protein to the neonate. Caseins constitute approximately 80% of all milk proteins and are made up of four major types: αs1-, αs2-, β-, and κ-casein. These types contribute 38%, 10%, 35%, and 15%, respectively, to the overall casein content [43]. Casein proteins are globular in shape, typically weighing between 19 and 25 kDa, and contain polar amino acid groups within their structure [35]. Casein solubility is highly sensitive to pH, temperature, and ionic strength, as well as to the presence of calcium and phosphate ions. At its isoelectric point (pI 4.6), casein becomes essentially insoluble, whereas its solubility increases as the pH shifts away from this value. At pH 5, the protein lacks net charge on its external functional groups, causing the molecules to aggregate and precipitate. As pH diverges from 5 in either direction, these functional groups become negatively or positively charged, increasing electrostatic repulsion and improving the dispersion of protein molecules. In biomedical applications, casein is preferred for the formulation of drug delivery systems, since it acts naturally as a carrier of micronutrients, such as calcium and phosphate. Moreover, it can form micelles with a hydrophobic core and a hydrophilic shell. This self-assembly enables the efficient encapsulation of hydrophobic compounds and protects them from oxidation, ultraviolet radiation, and temperature-related degradation [44].

Casein-based nanocarriers are used in research on natural compounds that may have cytotoxic effects against BC cells, such as MCF-7 or MDA-MB-231. Due to the presence of available amino (NH_2_) groups on the surface of the casein molecule, it has strong binding affinity for CUR. The interactions between the compound and the carrier were found to be hydrophobic and spontaneous in nature. The electrokinetic potential (ζ-potential) results for CUR entrapped in casein NPs indicate a high degree of colloidal stability, meaning that the particles maintain their dispersion in the aqueous medium. CUR, in its free form, is insoluble in water; therefore, encapsulation in casein-based nanocarriers improves its dispersibility and consequently makes the compound more bioavailable. Storage stability studies further demonstrated that the nanoformulation remains stable at 4 °C for at least 30 days, with unchanged particle size and ζ-potential and only about 10% drug leakage [99]. Some researchers reported that the lyophilized encapsulated form can be stable for even longer than 6 months when stored under refrigerated conditions [100,101].

Biological tests revealed the dose-dependent antiproliferative activity of both free and nanoencapsulated CUR. On the other hand, there was no significant cytotoxic activity of casein itself, suggesting its high biocompatibility. However, the nanocarrier formulation of CUR showed a highly significant reduction in the viability of cancer cells compared with free CUR. This can be clearly demonstrated by the lower IC50 values for the nanocarrier system. Furthermore, tests of free radical scavenging capacity showed that loading CUR into the carrier does not alter its antioxidant potential. For the encapsulate to be effective, it must be internalized into cells. Studies show that CUR in the carrier enters BC cells, particularly MCF-7 cells, to a significantly greater extent, which is consistent with viability studies. Casein NPs also demonstrate pH-dependent in vitro drug release. In an acidic environment, significantly more of the natural compound is released compared with physiological conditions (pH 7.4). This allows casein NPs to effectively deliver the compound to the predominantly acidic BC environment.

To further enhance specificity in cancer cell recognition, NPs can be modified by adding FA to their surface. As previously mentioned, this compound is strongly recognized by FR-α, which is overexpressed in breast tumors, but minimally present in normal tissues. This means that casein NPs exhibit marginal or no toxicity toward normal cells, demonstrating very high selectivity for BC cells [99,100,101].

Combinations of natural compounds and commonly used chemotherapeutic agents, such as paclitaxel (PTX), have also been incorporated into casein-based carriers. One such compound that demonstrated the potential of sensitizing is EGCG, which has demonstrated the potential to sensitize BC cells to PTX through the inhibition of glucose-regulated protein 78. The constructed system provided stepwise delivery, in which EGCG was released prior to PTX, thereby restoring the sensitivity of PTX-resistant MDA-MB-231 cells to PTX therapy [102]. Studies show that the encapsulation efficiency of natural compounds in casein NPs is remarkably high, enabling the delivery of substantial amounts of potentially cytotoxic agents directly to cancer cells [103].

Despite the limited number of animal studies investigating casein-based carriers in BC chemoprevention, Elbialy and Mohamed evaluated this approach using a transplantable, poorly differentiated carcinoma that originally developed spontaneously in mice as a breast tumor. In their work, proteins and polysaccharides were conjugated to produce CUR-loaded casein NPs coated with alginate and chitosan, forming a double polysaccharide layer (Alg-ch@CurCasNPs). Comprehensive in vivo results, including tumor inhibition rate, genotoxicity, and histopathological analysis, revealed that repeated oral administration of Alg-ch@CurCasNPs achieved superior therapeutic efficacy against Ehrlich carcinoma in mice compared with free CUR [104]. Animal studies also allow researchers to verify whether a given complex causes more damage in healthy tissue than in cancer tissue. Intravenous administration of a poly (lactic-co-glycolic acid) (PLGA)-caseinnanocarrier loaded with EGCG did not cause detectable organ toxicity, which was confirmed by the histopathological examination of major organs in Sprague–Dawley rats. Furthermore, in vivo pharmacokinetic analyses performed using the HPLC method demonstrated sustained and sequential release of the compound in plasma, indicating prolonged systemic circulation of the nanomedicine and improved compound availability compared with the free compound [105].

Even with these strengths, the application of casein as a vehicle for drug transport has some drawbacks. It tends to aggregate when placed in an aqueous environment close to its isoelectric point because of strong hydrophobic interactions. Additionally, its open tertiary structure and high proline content make it highly susceptible to pepsin digestion, potentially leading to rapid degradation of the carrier and premature release of encapsulated bioactive compounds [99].

### 3.4. Silk-Based Nanocarriers

Silk fibroin (SF) is one of the materials that has received extensive research attention due to its availability in nature, good physicochemical characteristics, excellent biocompatibility, and multifunctionality [106]. Silk proteins naturally occur in spiders, bees, and silkworms [45], but the most studied silk source is cocoon silk derived from the domesticated silkworm *B. mori* [46]. Silk fibers are composed of two types of proteins, namely fibroin and sericin, with fibroin serving as the main structural component, whereas sericin acts as the adhesive external layer. The SF structure is made up of two types of peptide chains: the heavy chain and the light chain, connected through a single disulfide bond located at the C-terminus of the heavy chain [45]. Naturally occurring silks are characterized by large molecular masses [46]. *B. mori* fibroin is a polymer protein with a molecular mass of about 415 kDa, comprising a heavy chain of 390 kDa (5242 amino acids) and a light chain of 26 kDa (246 amino acids) [47]. In silk fibers, fibroin constitutes roughly 75% (*w*/*w*) of the core, while sericin accounts for the remaining 25% as an external coating. Silk proteins have a set of unique features compared with other types of natural polymers. Depending on production and processing conditions, there are three conformational forms: Silk I, Silk II, and Silk III. Silk I is water-soluble and composed of random coil and α-helical structures, whereas Silk II contains a stable β-sheet structure and is water-insoluble. Silk III forms at the air–water interface [45] and possesses a triple-helical crystal structure [48]. Sericin is a group of water-soluble globular glycoproteins capable of inducing immune reactions and thus should be removed through degumming. Extraction of fibroin from the posterior glands of silkworm larvae in the fifth instar produces Silk I without degumming. Degumming of silk leads to the formation of water-insoluble fibroin (Silk II) that requires further processing to become soluble and thus is called regenerated fibroin [45]. The isoelectric point of SF is approximately 4 [46]. Raw fibroin is a semicrystalline protein composed of roughly 65% crystalline and 35% amorphous regions, providing mechanical strength and structural stability to the cocoon [106]. Silk I, which comprises many α-helices and hydroxyl groups, exhibits high water solubility and can be readily converted to Silk II by physical, chemical, or mechanical modifications. Silk II is hydrophobic and highly organized, with crystalline regions responsible for fibroin’s mechanical robustness; consequently, a higher Silk II content reduces fibroin solubility. The high dependence of silk solubility on its crystalline polymorphs provides the opportunity to tailor its solubility according to different process parameters. Up to 10% *w*/*v* aqueous solubility of silk I can be achieved, but above this level, the solution becomes viscous and undergoes gelation. Unlike other biopolymers, such as PLGA and PCL, the relatively high solubility of silk enables the preparation of drug delivery systems using fewer organic solvents [45]. Its amphiphilic nature allows the efficient encapsulation of both hydrophilic and hydrophobic agents [49].

Owing to all of these properties, SF is also used in delivery systems for compounds of natural origin in the chemoprevention of BC [107]. Studies have demonstrated that encapsulating CUR within a silk coating markedly enhances its cellular uptake in BC cell lines, including MDA-MB-231 and MCF-7, which translates into increased cytotoxic activity [108]. The SF shell provides an additional protective barrier that facilitates NP extravasation and prolongs intracellular retention, thereby reducing premature drug release and metabolic degradation. Prolonged intracellular residence improves drug availability and therapeutic performance [109]. Notably, this system achieved very high encapsulation efficiency (96%), whereas NPs prepared from a silk-chitosan blend showed lower values, suggesting that certain modifications beneficial for other protein carriers may not necessarily enhance silk-based systems [108]. In contrast, other findings indicate that incorporating chitosan into silk NPs can significantly improve the drug-loading efficiency and capacity, likely due to hydrogen bonding interactions between chitosan functional groups and CUR. Importantly, SF itself does not exhibit greater cytotoxicity than CUR alone, confirming its role as a biocompatible carrier rather than an active therapeutic agent [49]. The NPs also display a characteristic release profile, with an initial burst followed by sustained drug release over several days [108]. SF offers extensive opportunities for further functionalization owing to its amino acid composition. For instance, covalent conjugation with chondroitin sulfate enables targeting of the CD44 receptor, a transmembrane glycoprotein overexpressed in many cancer types, including BC. Functionalization with CD44-binding ligands improves targeting specificity and enhances the effectiveness of drug delivery systems [49]. Another promising approach involves combining SF with magnetic NPs, allowing externally guided targeting to tumor sites. This strategy enables the visualization of targeting, rapid localization, and magnetically induced heating to promote drug release while avoiding complex chemical surface modifications. Encapsulation of CUR in such magnetic silk carriers results in higher cellular uptake and enhanced cytotoxicity compared with the free compound [107]. Silk-based carriers have also been used to co-deliver natural compounds, such as CUR, with conventional chemotherapeutics, such as 5-fluorouracil. In these systems, intracellular ROS levels remain largely unchanged after exposure to free drugs but increase dramatically—up to tenfold—when cells are treated with drug-loaded NPs. Elevated ROS levels can trigger apoptosis through mitochondrial and endoplasmic reticulum stress pathways, contributing to enhanced anticancer activity [110].

In vivo studies further confirmed the therapeutic potential of these systems: tumor size decreased following the administration of drug-loaded NPs, whereas treatment with free drugs only resulted in a temporary slowing of tumor growth. Overall, these findings demonstrate that co-encapsulation of CUR and 5-fluorouracil in SF NPs, followed by percutaneous intratumoral administration, improved the therapeutic efficacy in a breast cancer model, specifically BALB/c mice bearing 4T1 tumors [110]. Although SF is an FDA-approved biopolymer that has been widely used in clinical applications, such as surgical sutures and tissue regeneration scaffolds, due to its biocompatibility, modifiable β-sheet structure, and versatile drug-loading capacity, SF NPs are still mainly investigated at the preclinical research stage. The main challenges limiting their clinical translation include batch-to-batch variability associated with degumming and regeneration procedures, the risk of immunogenic responses caused by residual sericin, inconsistent degradation behavior, and the lack of established GMP-compliant manufacturing processes [111]. Furthermore, *B. mori* full-length silk proteins are extremely difficult to recombinantly express in microorganisms due to their large size and highly repetitive amino acid sequences [47].

### 3.5. Comparative Profile of Animal-Derived Protein Nanocarriers

**Figure 1:**
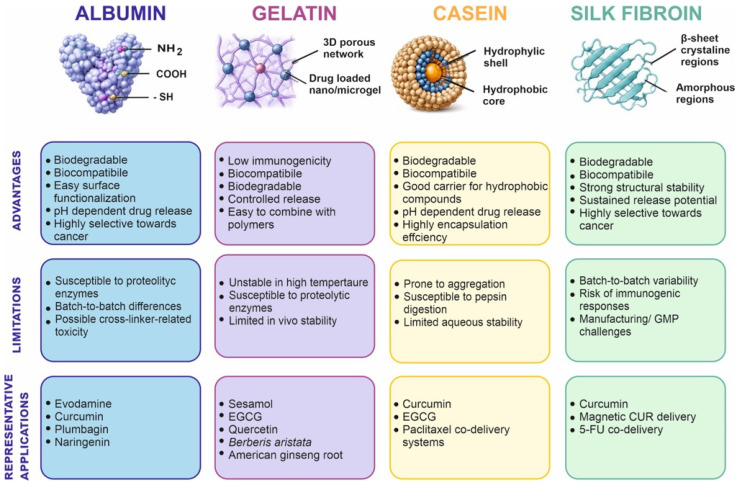
The major characteristics, advantages, limitations, and representative applications of animal protein-based nanocarriers discussed in this review. The figure compares albumin, gelatin, casein, and silk fibroin as animal-derived protein nanocarriers for natural compounds in BC chemoprevention and therapy. For each carrier, the key structural features, major advantages, current limitations, and representative encapsulated compounds or co-delivery systems are summarized. The protein icons included in the figure were generated using OpenAI’s ChatGPT (GTP-5.5).

Taken together, animal-derived protein nanocarriers, including albumin, gelatin, casein, and silk fibroin, represent a diverse group of delivery platforms with distinct structural and functional properties. Albumin-based systems are particularly advantageous for surface functionalization, tumor targeting, and improved systemic delivery, whereas gelatin-based carriers provide biodegradable matrices suitable for controlled and stimuli-responsive release. Casein nanocarriers are especially useful for the encapsulation of hydrophobic compounds due to their micelle-like organization, while silk fibroin-based systems offer high structural stability and sustained release potential. Despite these advantages, each platform also has specific limitations, including susceptibility to enzymatic degradation, batch-to-batch variability, aggregation, limited aqueous stability, or challenges related to large-scale manufacturing and clinical translation. A comparative summary of the major characteristics, advantages, limitations, and representative applications of animal protein-based nanocarriers discussed in this review is presented in Figure 1.

## 4. Plant-Derived Protein Nanocarriers

The use of plant protein nanocarriers is a relatively new and rapidly developing strategy in drug delivery. In response to the need for sustainability, cost-effectiveness, and environmental sustainability, plant protein-based NPs have emerged as promising alternatives to animal-derived biomaterials for the delivery of natural compounds in BC chemoprevention. Plant proteins such as zein, glutenin, and gliadin contain hydrophobic domains and/or amphiphilic regions that may support controlled drug release profiles. This intrinsic hydrophobicity and structural versatility allow these proteins to self-assemble into stable NPs without requiring the harsh chemical cross-linkers often needed in some animal-sourced systems. Plant proteins are economically viable, are not associated with the transmission of zoonotic pathogens, and contain a wide array of functional groups on their surface. The basic structural and physicochemical characteristics of these proteins are comprehensively summarized in Table 2.

### 4.1. Zein-Based Nanocarriers

Zein is a plant-derived hydrophobic protein primarily obtained from corn (*Zea mays* L.). Structurally, zein comprises four distinct fractions: α-, β-, γ-, and δ-zein, which differ in molecular weight, solubility, and amino acid sequence. The primary component is α-zein, accounting for approximately 80% of the total zein fraction and appearing as two distinct bands at 19 and 22 kDa. The minor fractions include γ-zein (10–20%; two bands at 18 and 27 kDa), β-zein (1–5%; 17 kDa), and δ-zein (1–5%; 10 kDa). Based on its amino acid composition, zein is classified as a prolamine. The presence of high proportions of non-polar amino acids, such as leucine (20%), proline (10%), and alanine (10%), makes it amphipathic and insoluble in pure water or pure ethanol, but highly soluble in alkaline solutions (pH ≥ 11.5), 70–95% aqueous ethanol solutions, and water–acetone mixtures. Additionally, its relatively high glutamine content (around 18%) may contribute to intermolecular interactions, while protonatable amino groups present in the protein structure are responsible for its positive surface charge under appropriate pH conditions. The protein’s isoelectric point is approximately 6.2 [113,114,115]. The absence of lysine and tryptophan, two indispensable amino acids, contributes to the poor nutritional quality of zein as a dietary protein. However, its intrinsic self-assembling nature, high biocompatibility, and amenability to chemical modification make zein an attractive material for drug delivery applications [124]. Crucially, zein’s secondary structure is highly dynamic and sensitive to environmental conditions. The α-helix content of zein progressively decreases in response to higher solvent polarity, elevated temperature, applied pressure, and pH-induced treatments, particularly under alkaline conditions. Such changes in the protein’s secondary structure play a critical role in determining the aggregation behavior, colloidal properties, and overall stability of zein NPs [113,114,115].

Zein-based NPs have attracted considerable interest as carriers for phytochemicals in BC chemoprevention, offering the potential to enhance their stability, delivery efficiency, and therapeutic efficacy. Algandaby et al. investigated the impact of piceatannol (PCT) encapsulated in zein nanostructures (PCT-ZN NSs) on the therapeutic potential of PCT against human breast cancer MCF-7 cells [125]. PCT is a natural polyphenolic stilbene with diverse pharmacological properties, including potent cytotoxic activity against various cancer cells [126]. PCT-ZN NSs exert enhanced anticancer effects in BC cells by improving cellular uptake and triggering apoptosis and oxidative stress. The formulation showed markedly higher cytotoxicity than free PCT, with IC50 values of 0.71 ± 0.06 µg/mL and 1.45 ± 0.12 µg/mL, corresponding to ~24-fold and ~21-fold increases in potency compared with free PCT (17.4 µg/mL). Cellular uptake was also significantly increased. In MCF-7 cells, uptake reached 34.2 ± 2.7% and 58.4 ± 3.4% at 2 and 4 h, compared with 16.2 ± 0.8% and 28.4 ± 1.9% for free PCT, while in MDA-MB-231 cells, it increased to 26.3 ± 1.2% and 52.9 ± 3.9%. Mechanistically, PCT-ZN NSs induced G0/G1 cell-cycle arrest and significantly increased apoptotic cell populations, accompanied by the upregulation of CASP3 (1.61-fold), p53 (1.86-fold), and Bax (1.32-fold), and the downregulation of Bcl-2 (2.13-fold) relative to free PCT. In addition, the nanospheres inhibited glutathione reductase activity by 50.49% (vs. 28.41% for PCT) and markedly increased ROS production, indicating strong oxidative stress-mediated apoptosis. Collectively, these effects highlight the multi-mechanistic anticancer action of PCT-ZN NSs, driven by the enhanced delivery and intracellular bioactivity of PCT [125]. Beyond PCT, zein-based nanocarriers have also been successfully applied for the delivery of other natural compounds, such as glycyrrhizic acid (GA). In this system, GA-loaded zein NPs (GA-LNPs) significantly enhanced cellular uptake, leading to greater intracellular accumulation of GA and increased exposure of MCF-7 BC cells to the drug, which translated into improved anticancer efficacy. In addition, the sustained-release profile of GA-LNPs prolonged drug availability at the cellular level, further amplifying cytotoxic effects. The incorporation of EDC as a crosslinker into the nanocarrier matrix may also protect GA from premature degradation, thereby preserving its structural integrity and biological activity. Consistent with observations for PCT-ZN NSs, encapsulation of GA within zein NPs enhanced its antioxidant potential and promoted a higher rate of apoptosis in MCF-7 cells. Collectively, these findings reinforce the role of zein-based nanocarriers as effective delivery platforms for natural bioactive compounds in cancer therapy [127].

Zein NPs demonstrate a pH-dependent, biphasic release profile that is beneficial for drug delivery in BC, as shown using β-carotene (βC) as a model compound. Under both physiological (pH 7.4) and acidic (pH ~1.2) conditions, βC-NPs showed a biphasic release profile characterized by an initial burst release followed by a sustained release over 96 h. The initial release was higher at acidic pH (~24%) than at pH 7.4 (~18%), which was attributed to weakly bound, surface-associated βC. During the first 4 h, cumulative release reached 39.9 ± 2.1% at pH 7.4 and 34.6 ± 2.7% at pH 1.2, while prolonged incubation resulted in approximately 90% and 83% total release at pH 7.4 and 1.2, respectively. Overall, βC-NPs displayed a slower and more sustained release pattern under both pH conditions, with enhanced release at physiological pH likely due to increased zein matrix solubility and gradual erosion, highlighting their suitability for controlled drug delivery applications [128]. In vivo studies are also being conducted, including those using BALB/c nude mice [129] and female Sprague–Dawley rats [130] to evaluate how zein-based encapsulation of natural compounds influences their anticancer activity. Encapsulation of isoliquiritigenin (ISL), a poorly water-soluble bioactive compound with strong anti-TNBC activity, within a zein-based nanocarrier significantly enhanced its anticancer efficacy. When orally administered to BALB/c nude mice, ISL-loaded ZLH nanoparticles (ISL-ZLH NPs; 40 mg/kg body weight) reduced tumor growth by 41.4% compared with free ISL, accompanied by a marked decrease in tumor weight relative to both blank nanoparticles and free drug treatment groups. In vitro, ISL@ZLH NPs also demonstrated significantly stronger antiproliferative and anti-colonogenic effects against TNBC cells than free ISL. Importantly, the NP formulation exhibited reduced cytotoxicity toward normal cells, indicating improved tumor selectivity and a more favorable therapeutic profile [129]. Another example involves female albino Swiss CD1 mice bearing Ehrlich ascites tumor cells injected into the left mammary fat pad, where intravenous administration of the carrier system significantly improved the anticancer efficacy of the loaded compounds, resulting in a 29.27% reduction in tumor volume compared with the control group [131].

Compared with animal-derived proteins, zein is more readily available and generally exhibits lower immunogenicity. However, its antigenic potential is influenced by the route of administration. Studies in mice have shown that intramuscular administration of zein elicits a systemic immune response. In contrast, oral delivery of zein NPs does not induce systemic immune activation but promotes systemic immune tolerance without triggering mucosal tolerance. Exposure to airborne zein particles may provoke type I hypersensitivity reactions, potentially leading to asthma. Furthermore, although zein is susceptible to hydrolysis by gastrointestinal proteases, it can still elicit immune reactions in individuals with celiac disease. This effect is attributed to the recognition of digested α-zein peptides by IgA antibodies present in patients with celiac disease, which identify these peptides as disease-associated antigens [124].

### 4.2. Glutenin-Based Nanocarriers

Glutenin constitutes one of the two main protein fractions forming gluten, a storage protein located in the endosperm of wheat grain [132]. The molecular weight of gluten proteins ranges from 30 to 90 kDa. Based on their solubility in aqueous alcohol solutions, gluten proteins are divided into two major fractions: monomeric gliadins and polymeric glutenins. Gliadins account for approximately 50–60% of gluten proteins and are soluble in aqueous alcohol solutions, whereas glutenins, representing approximately 40–50%, form the insoluble fraction under these conditions. Glutenin consists of two main groups of subunits: low-molecular-weight (LMW, 30–45 kDa) and high-molecular-weight (HMW, 67–88 kDa) subunits [133,134].

The structure of gluten is stabilized by disulfide bonds, both intramolecular and intermolecular, as well as by hydrogen bonds and hydrophobic interactions. These interactions determine the characteristic viscoelastic properties of gluten, which arise from the elasticity of glutenin and the viscosity of gliadins. Consequently, gluten proteins are of interest not only to the food industry, but also to the biomedical field, where they are being investigated as potential carriers for therapeutic agents [35,134].

Due to its considerable charge heterogeneity, glutenin does not possess a single characteristic isoelectric point. An approximate pI range of 6.5–7.0 is generally reported in the literature [116]; however, individual high-molecular-weight glutenin subunits (HMW-GS) exhibit substantial variation, with pI values ranging from 4.72 to 6.98. Among these, x-type and y-type subunits encoded by wheat glutenin genes can be distinguished. The y-type subunits are characterized by a more neutral character (pI 6.70–6.98), whereas most x-type subunits exhibit a more acidic character (pI 4.72–5.23) [117]. Functionally, glutenin is primarily responsible for the strength and elasticity of gluten. Its rheological properties arise largely from interactions with gliadins, and the relative proportion of these two fractions is one of the key factors determining the technological properties of gluten and the quality of bakery products [119]. Glutenin is also highly polymorphic and exhibits considerable allelic variability. Each wheat genotype synthesizes a specific set and quantity of glutenin subunits, and their expression depends on genetic factors as well as environmental conditions and technological processes applied during grain cultivation and processing [135].

The functional properties of glutenin have led to its industrial application, particularly in the production of vital wheat gluten and isolated wheat proteins used to improve the structure of bakery products and to enrich flours with low protein content [135]. In recent years, growing attention has also been directed toward the use of glutenin as a biomaterial for the development of controlled drug delivery systems, including nanocarriers with potential anticancer activity.

One example of such systems is FA-conjugated retinoic acid-loaded glutenin NPs (FA-RA-Glu NPs), designed for targeted drug delivery to BC cells expressing folate receptors. In vitro studies conducted on MCF-7 cells demonstrated a dose-dependent reduction in cell viability, with an IC50 value of 55.93 μg/mL. Moreover, this system exhibited good stability under physiological conditions and a sustained, pH-responsive release profile. Notably, retinoic acid release was greater under mild acidic conditions (pH 5.5), reaching 82.42 ± 0.75% after 33 h, compared with 71.36 ± 0.93% at pH 7.2, suggesting preferential drug release within the acidic tumor microenvironment. This targeted and controlled delivery resulted in enhanced anticancer activity against MCF-7 cells. Furthermore, the NPs disrupted cell membrane integrity and induced characteristic apoptotic changes, including cell shrinkage, nuclear fragmentation, and chromatin condensation. Additionally, increased cellular uptake of the NPs, induction of apoptosis, elevated ROS levels, and loss of mitochondrial membrane potential were also observed, confirming the anticancer potential of this system [136].

Another example involves glucose-conjugated glutenin NPs loaded with camptothecin (Glu-CPT-glutenin NPs). This approach exploits the altered metabolic profile of BC cells, which often exhibit increased glucose consumption and the overexpression of glucose transporters (GLUTs), particularly GLUT-1. After binding to GLUT-1, the glucose- modified nanocarrier can be rapidly internalized by tumor cells through receptor-mediated uptake, after which the transporter is recycled back to the cell membrane. Studies performed on MCF-7 cells revealed strong dose-dependent cytotoxicity, with an IC50 value of 18.23 μg/mL after 24 h of incubation. The mechanism of action involved mitochondrial damage, increased ROS production, and reduced mitochondrial membrane integrity, confirming the effectiveness of this therapeutic system [137].

Similar results were obtained for FA-CPT-PLGA-Glu NPs composed of PLGA and glutenin, functionalized with FA, and loaded with camptothecin. Studies on MCF-7 cells demonstrated high cytotoxic activity, with an IC50 value of 16.33 μg/mL. The presence of the folate ligand promoted efficient internalization by cancer cells, leading to increased ROS generation, inhibition of cell proliferation, and the induction of apoptosis associated with the activation of caspases-3 and -9 [118].

These findings indicate that glutenin may serve as a promising material for the development of anticancer drug nanocarriers. The systems developed to date enable the efficient delivery of active compounds, controlled drug release, and enhanced cytotoxicity against MCF-7 breast cancer cells [118,136,137]. However, the number of available studies remains limited, highlighting the need for further investigations to fully evaluate the potential of this biomaterial.

Despite the promising results obtained in laboratory studies, in vivo anticancer efficacy studies and clinical trials involving glutenin-based systems are still lacking. To date, it has only been demonstrated that glutenin NPs are biocompatible and stable under physiological conditions, and following intravenous administration in mice, they are distributed mainly to the liver, as well as to the spleen and kidneys, confirming their potential as drug carriers. However, no studies have yet evaluated their anticancer efficacy in in vivo models of BC [138].

The use of gluten proteins as carriers for therapeutic agents is also associated with several limitations. The most significant concern relates to immunological safety in susceptible individuals, including the risk of hypersensitivity reactions to gluten [139]. Another challenge is the limited stability of such systems within the gastrointestinal tract, as proteins are susceptible to degradation by digestive enzymes, which may compromise carrier integrity and reduce the efficiency of active compound delivery to the target site [140].

Additional limitations characteristic of protein-based delivery systems include considerable interindividual variability in bioavailability and absorption. This variability represents a major barrier to the translation of preclinical findings into clinical applications, making it difficult to predict therapeutic efficacy under in vivo conditions [141].

### 4.3. Gliadin-Based Nanocarriers

Gliadin is a major storage protein of wheat [142]. It consists of a single polypeptide chain with distinct hydrophilic and hydrophobic domains, which confer an amphiphilic character that is crucial for its functional properties [143]. On the basis of molecular weight and electrophoretic mobility, gliadins are classified into α- (25–35 kDa), β- (30–35 kDa), γ- (35–40 kDa), and ω-gliadins (55–70 kDa), although α- and β-gliadins are commonly grouped together due to their high sequence similarity. The proportions of these fractions vary depending on the wheat genotype and environmental conditions, with α/β- and γ-gliadins generally constituting the predominant forms [121].

Gliadin fractions differ in their structural organization. The α/β- and γ-gliadins contain significant proportions of α-helices and β-sheets, whereas ω-gliadin is characterized mainly by β-turn structures and lacks ordered α-helical and β-sheet regions. Moreover, the structural stability of gliadin varies among its fractions. While ω-gliadin is predominantly stabilized by hydrophobic interactions, α/β- and γ-gliadins derive their stability mainly from disulfide bonds and hydrogen bonding within their ordered secondary structures. These differences are reflected in their molecular conformations, with α/β-gliadins adopting compact globular structures and γ- and ω-gliadins exhibiting more elongated, rod-like conformations. In addition, the gliadin secondary structure is not solely determined by protein type, but is also influenced by environmental factors, including wheat cultivar, growth conditions, solvent composition, hydration level, and temperature.

The unique structural features of gliadin contribute to its low water solubility and high solubility in aqueous alcohol solutions. Together with its biodegradability, biocompatibility, mucoadhesive properties, and ability to self-assemble into NPs, these characteristics have made gliadin a promising carrier for the encapsulation and targeted delivery of bioactive compounds [122,143].

In the context of BC chemoprevention, this protein has been used as an encapsulation material for procyanidolic oligomers (PCOs), natural antioxidants derived from grape seeds with known anticancer potential. Using MCF-7 breast cancer cells, it was found that nanoformulated PCOs (PCOs-NF) exhibited greater growth-inhibitory activity than free PCOs, as reflected by a lower IC_50_ value (99.74 vs. 121.23 μg/mL). The effectiveness of PCOs-NF was confirmed through optical microscopic observations, which showed marked, concentration-dependent morphological changes. Unlike untreated cells, which displayed clearly visible nuclei and preserved morphology, the PCOs-NF-exposed cells developed a rounded morphology, aggregated into irregular clumps, and showed a progressive loss of cell viability. Cell death was evident at 31.25 μg/mL, increased substantially at 62.5 μg/mL, and at 125 μg/mL, extensive cell rupture and death were observed, with an effect comparable to that of cisplatin. These findings indicate that gliadin-based nanoencapsulation can enhance the anticancer activity of natural antioxidants against BC cells [123]. Additional examples of natural compounds delivered using gliadin-based nanocarriers include resveratrol [144] or CUR [145]. Despite their potential as drug delivery vehicles, gliadin-based NPs are often unstable, exhibit a tendency to aggregate, and show relatively low encapsulation efficiency. To overcome these limitations, they can be coated with additional materials, such as lipids or polysaccharides, forming core–shell nanostructures in which gliadin constitutes the core and the stabilizing polymer forms the outer shell. Among these materials, pectin is a promising coating agent due to its excellent stability under a wide range of environmental conditions and its ability to improve NP stability through protein–polysaccharide complexation. Using this approach, Banaee et al. developed a pectin-coated gliadin nanocomposite (G-P) for CUR delivery with the aim of improving its aqueous dispersibility and bioavailability. The release profile of CUR was evaluated in phosphate-buffered saline (PBS, pH 7.4) and sodium acetate buffer (pH 5.0). In both media, an initial burst release was observed during the first 60 min, followed by a slower and more sustained release phase. After 240 min, approximately 83% of the encapsulated CUR had been released at pH 7.4, whereas 87% was released at pH 5.0. The observed pH responsiveness is particularly relevant for cancer therapy because many tumor microenvironments exhibit acidic extracellular pH values (approximately 5–6), potentially enabling enhanced drug release at the tumor site while limiting premature release under physiological conditions. The improved delivery performance of Cur-G-P was reflected in its anticancer activity against MCF-7 breast cancer cells. Increasing the CUR concentration from 0.06 to 2 mg/L significantly enhanced the cytotoxic effect of the nanocomposite, reducing the cell viability from 69.97 ± 4.01% to 19.98 ± 2.25% after 48 h of treatment. In comparison, free CUR reduced the cell viability from 88.89 ± 1.10% to only 38.40 ± 2.00% over the same concentration range. Consistent with these findings, the IC50 value of Cur-G-P was approximately fourfold lower than that of free CUR, indicating substantially greater therapeutic potency. Morphological observations further supported the cytotoxicity results. After 48 h of exposure to Cur-G-P, MCF-7 cells exhibited pronounced structural alterations, including cell detachment, loss of their normal morphology, cell rounding and enlargement, and increased cytoplasmic roughness. In contrast, untreated cells remained adherent and maintained their typical morphology, indicating that the nanocomposite effectively induced cellular damage and growth inhibition [146].

The potential of gliadin-based nanocarriers has also been demonstrated in vivo. In a BALB/c mouse BC model, hyaluronic acid-functionalized, usnic acid-loaded gliadin NPs (HA-UA-GNPs) exhibited superior antitumor activity compared with both non-targeted UA-GNPs and free usnic acid. The enhanced therapeutic efficacy was attributed to CD44-mediated targeting, which resulted in greater cellular uptake and cytotoxicity of the HA-modified formulation. Histopathological analyses further revealed a greater extent of tumor necrosis in mice treated with HA-UA-GNPs, supporting the effectiveness of the targeting strategy. These findings highlight that surface-functionalized gliadin NPs can significantly improve the delivery and antitumor performance of natural compounds in in vivo models of BC [147].

Despite the promising results reported for gliadin-based nanocarriers in the delivery of natural anticancer compounds, several limitations currently hinder their translation into clinical applications. First, the available evidence is largely restricted to in vitro studies, with only a limited number of in vivo investigations and an absence of clinical trials. Consequently, the long-term safety, pharmacokinetic behavior, biodistribution, and therapeutic efficacy of gliadin-based formulations in humans remain insufficiently characterized. In addition, the physicochemical properties of gliadin can pose formulation challenges. Owing to its highly aggregated structure and extensive intermolecular disulfide cross-linking, glutenin exhibits poor solubility in aqueous media, which may complicate NP preparation and drug loading. Another important consideration is the potential immunogenicity associated with wheat-derived proteins. Although NP formulations may reduce direct protein exposure, further studies are required to evaluate the risk of immune responses and allergenicity, particularly following repeated administration. Therefore, while gliadin represents a promising plant-based carrier for BC chemoprevention, additional preclinical and clinical studies are needed to fully establish its safety, efficacy, and translational potential.

### 4.4. Comparative Profile of Plant-Derived Protein Nanocarriers

**Figure 2:**
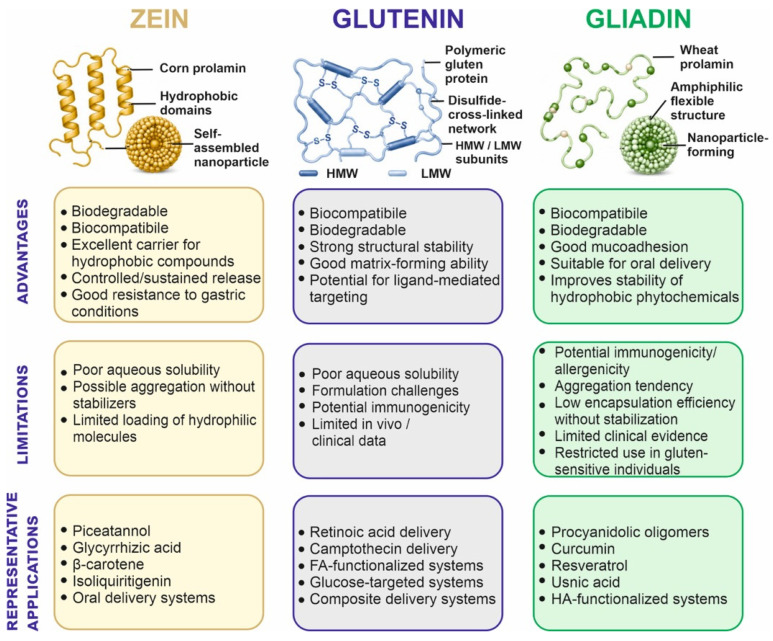
Comparison of plant protein-based nanocarriers used for the delivery of natural compounds in BC chemoprevention. The figure presents a schematic comparison of zein-, glutenin-, and gliadin-based nanocarriers, highlighting their key structural characteristics, advantages, limitations, and representative applications in the encapsulation and delivery of natural bioactive compounds for BC chemoprevention and therapy. The protein icons included in the figure were generated using OpenAI’s ChatGPT (GTP-5.5). LMW—low-molecular-weight subunit; HMW—high-molecular-weight subunit.

From a comparative perspective, plant-derived protein nanocarriers, including zein, glutenin, and gliadin, represent a promising and increasingly explored group of delivery platforms for natural bioactive compounds in BC chemoprevention. Zein-based systems are particularly advantageous due to their hydrophobic character, intrinsic self-assembly ability, and suitability for the encapsulation of poorly water-soluble phytochemicals. Glutenin-based nanocarriers offer the possibility of ligand-mediated targeting and controlled release, largely owing to their polymeric structure and the presence of functional groups involved in intermolecular interactions. Gliadin-based systems, in turn, combine amphiphilic properties, biodegradability, and the ability to form nanoparticles that can be further stabilized or functionalized using polysaccharides, lipids, or targeting ligands. Despite these advantages, plant protein-based nanocarriers also present important limitations, including poor aqueous solubility, aggregation tendency, susceptibility to enzymatic degradation, potential immunogenicity or allergenicity, and limited in vivo and clinical evidence. A comparative profile of the major characteristics, advantages, limitations, and representative applications of plant protein-based nanocarriers discussed in this review is presented in Figure 2.

## 5. Ferritin-Based Nanocarriers as Cross-Origin Protein Nanoplatforms

In addition to animal- and plant-derived protein nanocarriers, ferritin represents a distinct cross-origin protein nanoplatform, as ferritin homologues occur in animals, humans, microorganisms, and plants. Therefore, ferritin-based nanocarriers are discussed separately, with particular emphasis on their structural features, intrinsic self-assembly, and potential applications in the targeted delivery of natural compounds for BC chemoprevention.

Ferritins are highly conserved proteins that are widely distributed across diverse organisms, ranging from archaea to humans. They are also found in viruses and mycoplasmas. The ferritin family includes ferritin itself, bacterioferritin, and DNA-binding mini-ferritin. Plant ferritin is sometimes considered a separate member of the family and is called phytoferritin [148,149]. All ferritin subunits share a length of approximately 200 amino acids and a four-helix bundle folding motif as common structural features [149]. The overall structure of this approximately 500 kDa protein complex consists of 24 subunits, typically composed of heavy and light chains, which can self-assemble into a highly symmetric nanocage with a particle size of 12 nm and an internal diameter of 8 nm [150]. Ferritin has an isoelectric point between pH 4.6 and 5.0 [151]. Its physiological function is to protect cells from damage caused by the Fenton reaction and to act as an iron storage system. Table 3 provides an overview of the structural and physicochemical properties of ferritin.

Due to their high stability and ease of preparation in vitro, ferritins represent an intriguing system for nanobiotechnology and drug delivery applications [149]. Paclitaxel encapsulated in ferritin exhibited enhanced cytotoxicity against tumor cells, including the MCF-7 breast cancer cell line [152], which was also demonstrated in a BALB/c mouse model [153]. This is an example of insoluble drug delivery via ferritin, with the specific targeting of tumor cells helping to alleviate the adverse effects of this chemotherapeutic agent [149].

Importantly, ferritins are also used for the delivery of compounds of natural origin, not only chemically synthesized drugs. CUR encapsulated in a self-assembling human apoferritin nanocarrier has several advantages over the free compound. The nanoformulation improves CUR solubility and enhances its uptake by BC cells (MDA-MB-231 and MDA-MB-468), which together contribute to the higher cytotoxicity observed for NP-bound CUR compared with free CUR. CUR is a pleiotropic agent, meaning that it interacts with several molecular targets simultaneously and influences various signaling cascades inside the cell. Besides its ability to inhibit the viability of the above-mentioned BC cells, CUR NPs were also found to decrease the phosphorylation of Akt protein to the same level as that observed for free CUR, which disrupts the functioning of the PI3K/Akt signaling pathway and inhibits cell growth and cycle progression. Moreover, it was demonstrated that this NP formulation enhanced the effect of CUR on cell-cycle progression, resulting in an accumulation of cells in the G2/M or G0/G1 phases. Another important effect of CUR is its ability to enhance the sensitivity to anticancer agents through a variety of mechanisms. To determine whether CUR encapsulated within the ferritin shell retains this activity, it was used in combination with DOX [154], an anticancer drug that is known for inducing chemoresistance in BC [155,156]. The viability assays showed that the encapsulated formulation sensitized cells to DOX to a much greater extent than free CUR in solution. Sensitization to this drug may occur via several mechanisms, one of which involves alterations in MDR proteins such as P-glycoprotein (P-gp) and multidrug-resistant protein 1 (MRP1). These mechanisms may either inhibit drug efflux or downregulate the expression of MDR genes. To assess the function of these transporters, specific inhibitors were used; verapamil inhibited P-gp, while MK571 reduced MRP1 activity. The sensitivity to inhibitors was evaluated by determining the remaining fluorescent signal using flow cytometry. The results confirmed that CUR in ferritin NPs may induce the sensitization of BC cells, potentially through a mechanism involving those two proteins and these two transporters [154]. This is not an isolated example of the enhanced effect of a natural compound when encapsulated in ferritin. Significantly higher, concentration-dependent growth inhibition was also observed when Jerantinine A was encapsulated in horse spleen ferritin. This O-acetyl derivative of the *Aspidosperma* alkaloid demonstrated growth inhibition in several BC cell lines (SKBR-3, MDA-MB-231, MDA-MB-468, BT-474, MCF-7) even at low concentrations, whereas the compound itself required a much higher concentration to achieve similar effects. Furthermore, it was shown that the carrier alone did not affect growth or viability in any cell line, suggesting high ferritin biocompatibility [157]. It has also been shown that more than one compound can be co-encapsulated within the ferritin shell. Such co-encapsulation and coadministration of CUR and quercetin resulted in synergistically greater viability inhibition, as well as an increased percentage of cells in early apoptosis and late apoptotic/dead cells [158]. Ferritin can tolerate a broad range of pH and keep its spherical hollow shape intact. Extreme pH conditions (2 to 3 or 10 to 12) cause the spherical shell to dissociate into subunits, while at physiological pH, the nanocage reassembles into its original shape [148,159,160]. This property has been used, for example, for the encapsulation of quercetin and CUR into the ferritin cavity [158]. Moreover, ferritin is also able to withstand high temperatures up to 75 °C for 10 min. It is also highly stable in the presence of various denaturants [160].

As in the case of albumin, ferritin can be conveniently functionalized through its structural units as well as through conjugation with a variety of molecules, including small proteins or peptides. This can be accomplished through simple modifications to the primary sequence of ferritin while retaining the ability of the NP cage to self-assemble. Surface functionalization can introduce beneficial features, such as cellular targeting ability, whereas the truncation of peptide units can facilitate passive diffusion of the cargo molecules. However, this could come at the cost of less controlled drug release [161]. The use of PASylation, which involves the attachment of short, hydrophilic, uncharged sequences consisting of proline, alanine, and serine to the N-terminus or C-terminus of protein-based drugs, further enhanced the anticancer properties of wogonin (Wog), a natural flavonoid isolated from the root extract of *Scutellaria baicalensis*. The physical characteristics of PAS peptides mimic those of PEGs while avoiding many of their disadvantages, including expensive synthesis and cellular vacuolation. Additionally, the viscosity and hydrophilicity of PAS polymers are greater than those of comparably sized PEG molecules, which may facilitate drug delivery. PASylation primarily improves the stability of ferritin-based carriers and their cargo. Drug release profiles differed markedly between PASylated human H chain ferritin (HFtn)-Wog and non-PASylated HFtn-Wog NPs. Approximately 50% of Wog was released from HFtn-Wog NPs within 10 days, whereas PASylated HFtn-Wog, particularly PAS30-HFtn-Wog, showed substantially greater stability, with less than 15% release over two months at 4 °C. In addition, NP stability at 4 °C was significantly higher than at 37 °C, consistent with the known susceptibility of protein-based NPs to depolymerization and deactivation at elevated temperatures [162].

A major advantage of ferritin as a drug carrier in BC therapy is its high selectivity toward tumor cells, while non-tumorigenic breast epithelial cells show minimal uptake [158]. One proposed explanation for this preferential accumulation is the increased iron demand of tumors, which promotes ferritin uptake [160]. Another important mechanism underlying this selectivity is the interaction between ferritin and transferrin receptor 1 (TFR1), which mediates cellular internalization. TFR1 is expressed at low levels in normal cells but is strongly overexpressed in many cancers, including BC [157,163].

In vivo studies using BALB/c mice have also shown that naturally occurring compounds, such as CUR, when encapsulated within a protein shell and administered intravenously, exhibit enhanced anticancer activity. A breast tumor growth inhibition of approximately 60% has been observed compared with the untreated control group. The use of these carriers appears to be safe, as the NPs did not cause significant body weight loss, among other assessed safety parameters [164,165].

Despite its many advantages, ferritin also presents several limitations as a drug delivery carrier. One important drawback is the relatively high production cost associated with protein-based nanocarriers, including ferritin, which may limit large-scale manufacturing and clinical translation [18]. In addition, ferritin nanocages often exhibit relatively low drug-loading capacity compared with some synthetic nanocarriers, which may restrict the achievable therapeutic dose and require the further optimization of loading strategies. Another major limitation is the limited availability of clinical data. Most studies investigating ferritin-based delivery systems, particularly those involving natural compounds, remain at the in vitro stage, with only a small number of in vivo studies conducted so far. Consequently, further preclinical and clinical research is necessary to fully evaluate the safety, efficacy, and long-term pharmacological performance of ferritin-based drug delivery systems.

**Figure 3:**
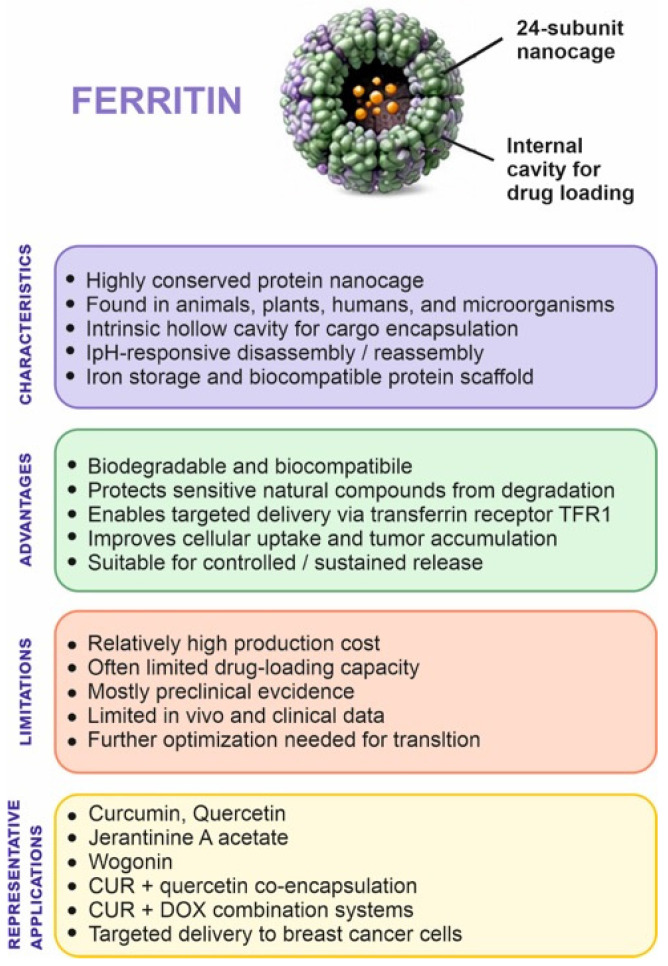
Major characteristics, advantages, limitations, and representative applications of ferritin-based nanocarriers discussed in this review. The figure presents a schematic overview of ferritin as a cross-origin protein nanoplatform for the encapsulation and targeted delivery of natural bioactive compounds in BC chemoprevention and therapy. It highlights the nanocage structure of ferritin, its self-assembly properties, pH-dependent disassembly and reassembly, receptor-mediated cellular uptake, major advantages, current limitations, and representative applications in the delivery of natural compounds and combination systems. The protein icon included in the figure was generated using OpenAI’s ChatGPT (GTP-5.5).

Considering these features, ferritin-based nanocarriers represent a unique cross-origin protein platform with high structural stability, intrinsic self-assembly capacity, and the ability to encapsulate or co-encapsulate natural bioactive compounds. Their nanocage architecture, pH-dependent disassembly and reassembly, and receptor-mediated uptake, particularly through TFR1, make them promising systems for targeted delivery in BC chemoprevention and therapy. However, further optimization of drug-loading capacity, manufacturing scalability, long-term safety, and clinical validation is required before ferritin-based systems can be translated into routine biomedical applications. The major structural features, advantages, limitations, and representative applications of ferritin-based nanocarriers discussed in this review are summarized in Figure 3.

## 6. Future Perspectives and Conclusions

Plant-based natural products have long been recognized as a vital source of anticancer and chemopreventive agents. Studies have demonstrated that compounds such as CUR, EGCG, quercetin, and other phytochemicals exert cytotoxic and chemopreventive effects against BC. Although many phytochemicals have shown promising activity against BC in preclinical studies, their broader clinical application remains constrained by suboptimal pharmacokinetic behavior and insufficient maintenance of therapeutically relevant levels at the target site. These limitations are particularly important in the context of chemoprevention, where long-term safety, sustained exposure to bioactive compounds, and selective activity against early carcinogenic changes are essential.

Importantly, different protein-based systems exhibit distinct structural and functional properties that may influence their suitability for specific biomedical applications. Albumin-based nanocarriers are particularly attractive because of their high biocompatibility and intrinsic tumor-targeting ability, whereas ferritin NPs enable efficient cellular uptake through receptor-mediated mechanisms. Gelatin and casein systems provide favorable biodegradability and controlled release properties, while silk-based carriers exhibit exceptional mechanical stability and prolonged drug release potential. Thus, animal-derived proteins, plant-derived proteins, and ferritin-based nanoplatforms should be considered complementary rather than interchangeable delivery systems, because their usefulness depends on the dominant physicochemical or biological limitation of the encapsulated compound.

From a practical formulation perspective, the optimal protein nanocarrier should be selected according to the key limitation of the natural compound. Thus, carriers should be chosen based on whether the main challenge is poor solubility, rapid degradation, insufficient stability, limited bioavailability, the need for controlled release, or insufficient tumor selectivity. In this context, no single protein carrier can be considered universally superior; rather, each system should be matched to the physicochemical properties of the encapsulated compound, route of administration, target tissue, and intended chemopreventive or therapeutic application.

**Figure 4:**
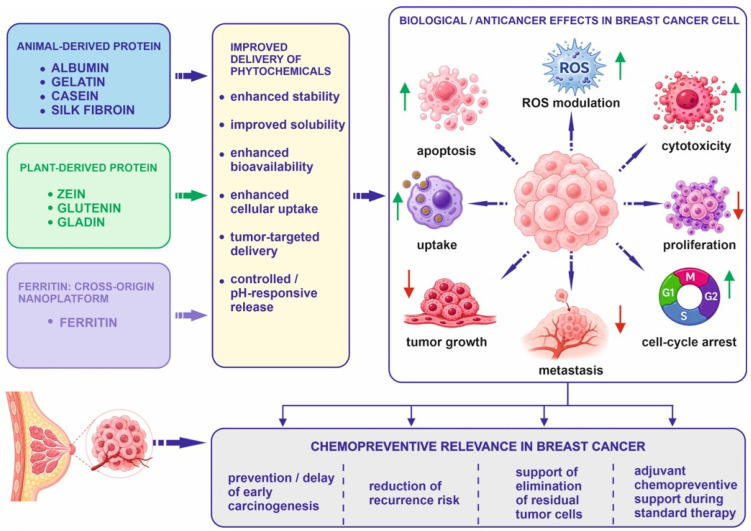
Chemopreventive and anticancer roles of protein-based nanocarriers in breast cancer. The figure presents a schematic overview of the animal-derived, plant-derived, and ferritin-based protein nanocarriers discussed in this review and their role in improving the delivery of natural bioactive compounds. These systems may enhance stability, solubility, bioavailability, cellular uptake, tumor-targeted delivery, and controlled release, thereby supporting key anticancer and chemopreventive effects in breast cancer, including apoptosis induction, oxidative stress modulation, cell-cycle arrest, tumor growth inhibition, recurrence risk reduction, and adjuvant support of standard therapy. The icons included in the figure were generated using OpenAI’s ChatGPT (GTP-5.5). Legend: ↑ activation or increase; ↓ inhibition or decrease.

Beyond their role as delivery vehicles, protein-based nanocarriers may contribute to BC chemoprevention at several biologically relevant stages, as summarized in Figure 4. By improving the stability and bioavailability of phytochemicals, they may support long-term exposure to compounds capable of modulating oxidative stress, apoptosis, cell-cycle progression, tumor growth, and metastatic processes. In this context, these systems may be relevant not only for inhibiting tumor growth, but also for preventing or delaying the early stages of carcinogenesis, reducing recurrence risk, supporting the elimination of residual tumor cells, and serving as adjuvant chemopreventive tools in individuals at increased risk of BC. This chemopreventive perspective should be strengthened in future studies, because most available data still focus on cytotoxicity against established cancer cell lines rather than on tumor initiation, pre-invasive lesions, residual disease, or recurrence prevention.

However, future development should move beyond material-oriented optimization and more directly address biological questions relevant to BC prevention and disease control. These include determining whether encapsulated phytochemicals can modulate early carcinogenic pathways, evaluating their effects on the tumor microenvironment and immune response, identifying BC subtypes that may benefit most from specific nanocarrier systems, and assessing their capacity to reduce recurrence after standard therapy. Advanced 3D cultures, organoid models, patient-derived models, and well-designed in vivo chemoprevention studies will be particularly important for bridging the gap between formulation development and clinically meaningful biological outcomes.

Nevertheless, several challenges still limit the clinical translation of protein-based nanocarriers. These include difficulties associated with large-scale production, high purification costs, protein instability during storage, batch-to-batch variability, limited reproducibility, and the necessity to optimize formulations individually for different proteins and bioactive compounds. Additional limitations include incomplete understanding of long-term toxicity, insufficient pharmacokinetic and biodistribution data, and the relatively small number of in vivo and clinical studies evaluating the efficacy of protein-based systems in BC patients. For chemopreventive applications, these challenges are particularly important because formulations intended for long-term or repeated administration must demonstrate excellent safety, predictable degradation, minimal immunogenicity, and stable biological activity over time.

In conclusion, protein-based NPs represent highly promising multifunctional platforms for improving the chemopreventive and therapeutic potential of natural compounds in breast cancer. The key conclusion from the current evidence is that their effectiveness depends on rational carrier selection. Albumin and ferritin are particularly suitable when tumor targeting and receptor-mediated uptake are required; gelatin and casein are useful when controlled or pH-responsive release is needed; silk fibroin is advantageous for sustained release and structural stability; and zein, glutenin, and gliadin provide promising plant-derived alternatives for the encapsulation and delivery of hydrophobic phytochemicals, especially when sustainability and non-animal origin are important considerations. Future progress in this field will depend on interdisciplinary collaboration between bioorganic chemistry, macromolecular chemistry, materials science, nanomedicine, molecular oncology, and translational medicine. In our view, the next stage of research should prioritize the direct validation of chemopreventive efficacy in biologically relevant BC models, including models of early carcinogenesis, high-risk conditions, tumor recurrence, and adjuvant therapy. Such studies will be essential to determine whether protein-based nanocarriers can move from promising experimental delivery platforms toward practical tools for BC prevention, recurrence reduction, and supportive chemopreventive therapy.

## Figures and Tables

**Table 1:** Basic properties of the animal-derived proteins used as nanocarriers of natural compounds in breast cancer chemoprevention.

Protein	Molecular Weight (kDa)	Isoelectric Point	Water-Solubility	Structural Features	Refs.
Albumin	HSA: 66.5 BSA: 69	HSA and BSA: 4.7	Yes	Globular protein; HSA: one polypeptide chain made up of 585 amino acid residues with a high number of cysteine and low content of tryptophan residues; BSA: contains multiple disulfide bonds that act as intrinsic cross-linking.	[33,34,35,36,37]
Gelatin	Type A: 90–100 Type B: 40–90	Type A: 9 Type B: 5	Yes	Fibrous protein; Polypeptide chains that consist of 19 amino acids with a repeating (Gly-X-Pro)_n_ motif with glycine as the dominant residue, and X is often proline or hydroxyproline.	[38,39,40,41]
Casein	19–25	4.6	Yes	Globular protein; αs1- and αs2-caseins: unfolded with extended coil; β- and κ-caseins: molten globule-like; all contain N-terminal signal peptides and Pro-Gln–rich C-terminal regions forming a potential amorphous calcium phosphate-binding motif.	[35,42,43,44]
Silk	415	4	Silk I Yes Silk II No Silk III forms at the water–air interface	SF fibrous protein and sericin globular protein; three conformational forms: Silk I is composed of random coil and α-helical structure; Silk II contains a stable β-sheet structure; Silk III has a triple helical crystal structure.	[45,46,47,48,49]

Legend: BSA—bovine serum albumin; HSA—human serum albumin.

**Table 2:** Basic properties of plant derived-proteins used as nanocarriers of natural compounds in breast cancer chemoprevention.

Protein	Molecular Weight (kDa)	Isoelectric Point	Water-Solubility	Structural Features	Refs.
Zein	α-zein: 19–22 β-zein: 17 γ-zein: 18–27 δ-zein:10	6.2	No	Rich in hydrophobic residues (20% Leu, 10% Pro, 10% Ala); environment-sensitive, highly dynamic secondary structure; 9–10 antiparallel α-helices linked via glutamine-rich loops.	[112,113,114,115]
Glutenin	LMW: 30–45 HMW: 67–88	6.5–7.0	No	Interchain disulfide bonds; native glutenins are composed of a backbone formed by HMW subunit polymers and of LMW subunit polymers branched off from HMW subunits	[35,116,117,118,119]
Gliadin	α-gliadin: 25–35, β-gliadin: 30–35, γ-gliadin: 35–40, ω-gliadin: 55–70	5.5	Low	α/β- and γ-gliadins rich in α-helices and β-sheets; ω-gliadin lacks ordered regions, consisting predominantly of β-turns; α/β- and γ-gliadins stabilized by intramolecular disulfide links and hydrogen bonding; ω-gliadin driven primarily by hydrophobic interactions; compact, globular structures in α/β-gliadins versus elongated, rod-like conformations in γ- and ω-gliadins.	[120,121,122,123]

Legend: LMW—low-molecular-weight subunit; HMW—high-molecular-weight subunit.

**Table 3:** Basic properties of ferritin as nanocarrier of natural compounds in breast cancer chemoprevention.

Protein	Molecular Weight (kDa)	Isoelectric Point	Water-Solubility	Structural Features	Refs.
Ferritin	500	Between 4.6 and 5.0	Yes	Globular protein; Consists of 24 subunits of light and heavy chains; Built of 200 amino acids and a four-helix bundle folding motif.	[149,150,151]

## Data Availability

No new data were created or analyzed in this study. Data sharing is not applicable to this article.

## Abbreviations

The following abbreviations are used in this manuscript:

Alg-ch@CurCasNPs	CUR-loaded casein NPs coated with alginate and chitosan, forming a double polysaccharide layer
BA/GCD	Betulinic acid encapsulated in γ-cyclodextrin
BA/GGNPs	Betulinic acid encapsulated in gelatin–γ-cyclodextrin nanoparticles
BC	Breast cancer
BET	Betanin
BSA	Bovine serum albumin
βC	β-carotene
CUR	Curcumin
DOX	Doxorubicin
EGCG	Epigallocatechin gallate
ENPs	Evodiamine-loaded nanoparticles
FA	Folic acid
FA-RA-Glu NPs	FA-conjugated RA-loaded glutenin NPs
FDA	Food and Drug Administration
FRα	Folate receptor alpha
GA	Glycyrrhizic acid
GA-LNPs	GA-loaded zein NPs
GLN-BA	Gelatin-based lipid nanocarriers encapsulating *Berberis aristata* extract
Glu-CPT-glutenin NPs	Glucose-conjugated glutenin NPs loaded with camptothecin
GLUT	Glucose transporters
GNPs	Gelatin nanoparticles
GONs	Oleic acid-conjugated gelatin NPs
HA-UA-GNPs	Hyaluronic acid-functionalized usnic acid-loaded gliadin NPs
HFtn	Human H chain ferritin
HMW	High-molecular-weight
HSA	Human serum albumin
ISL	Isoliquiritigenin
LMW	Low-molecular-weight
MDR	Multidrug resistance
MRP1	Multidrug-resistant protein 1
NPs	Nanoparticles
PCOs	Procyanidolic oligomers
PCOs-NF	Nanoformulated PCOs
PCT	Piceatannol
PCT-ZN NSs	Piceatannol in the form of zein nanostructures
PEG	Polyethylene glycol
P-gp	P-glycoprotein
PLGA	Poly-L-lactide-co-glycolic acid
Ptx	Paclitaxel
ROS	Reactive oxygen species
SF	Silk fibroin
TFR1	Transferrin receptor 1
Wog	Wogonin
